# Identification of Lipases Involved in PBAN Stimulated Pheromone Production in *Bombyx mori* Using the DGE and RNAi Approaches

**DOI:** 10.1371/journal.pone.0031045

**Published:** 2012-02-16

**Authors:** Mengfang Du, Xinming Yin, Songdou Zhang, Bin Zhu, Qisheng Song, Shiheng An

**Affiliations:** 1 College of Plant Protection, Henan Agricultural University, Zhengzhou, People's Republic of China; 2 Division of Plant Sciences, University of Missouri, Columbia, Missouri, United States of America; Baylor College of Medicine, United States of America

## Abstract

**Background:**

Pheromone biosynthesis activating neuropeptide (PBAN) is a neurohormone that regulates sex pheromone synthesis in female moths. *Bombyx mori* is a model organism that has been used to explore the signal transduction pattern of PBAN, which is mediated by a G-protein coupled receptor (GPCR). Although significant progress has been made in elucidating PBAN-regulated lipolysis that releases the precursor of the sex pheromone, little is known about the molecular components involved in this step. To better elucidate the molecular mechanisms of PBAN-stimulated lipolysis of cytoplasmic lipid droplets (LDs), the associated lipase genes involved in PBAN- regulated sex pheromone biosynthesis were identified using digital gene expression (DGE) and subsequent RNA interference (RNAi).

**Results:**

Three DGE libraries were constructed from pheromone glands (PGs) at different developed stages, namely, 72 hours before eclosion (−72 h), new emergence (0 h) and 72 h after eclosion (72 h), to investigate the gene expression profiles during PG development. The DGE evaluated over 5.6 million clean tags in each PG sample and revealed numerous genes that were differentially expressed at these stages. Most importantly, seven lipases were found to be richly expressed during the key stage of sex pheromone synthesis and release (new emergence). RNAi-mediated knockdown confirmed for the first time that four of these seven lipases play important roles in sex pheromone synthesis.

**Conclusion:**

This study has identified four lipases directly involved in PBAN-stimulated sex pheromone biosynthesis, which improve our understanding of the lipases involved in releasing bombykol precursors from triacylglycerols (TAGs) within the cytoplasmic LDs.

## Introduction

In most lepidopteran insects, females release volatile blends of sex pheromones to attract males for mating. Thus, sex pheromones serve as the key mediators of sexual communications for population propagation. These sex pheromone blends are synthesized and released by pheromone gland (PG) located between the eighth and ninth abdominal segments of females. Sex pheromones are derived from acetyl-CoA through fatty acid synthesis, desaturation, and chain-shortening reactions followed by the reductive modification of the carbonyl carbon [1].

The synthesis and release of sex pheromones in most moths are triggered by pheromone biosynthesis activating neuropeptide (PBAN), a 33-amino-acid peptide amidated at the C-terminus. PBAN was first identified in *Helicoverpa zea* and *Bombyx mori* and thereafter has been isolated from a variety of species [2], [3]. PBAN, produced by the subesophageal ganglion after adult emergence, acts directly on the PG cells to activate the various steps of the sex pheromone biosynthesis pathway [4]. In fact PBAN regulated pheromone synthesis in a species-dependent manner. In the genus *Helicoverpa*, PBAN appears to accelerate enzymatic steps leading to fatty acid synthesis, most likely acetyl-CoA carboxylase activity [5], [6]. In constrast, in *B. mori*, PBAN was found to directly regulate the terminal step of fatty acyl reduction [7], [8]. Most importantly, cAMP is found to be required for *Helicoverpa* PBAN signaling, however not involved in *Bombyx* PBAN signaling [9].

PBAN actions in *B. mori* have been investigated in impressive detail and the associated functional proteins involved in sex pheromone synthesis and release have been identified and characterized [4], [9]. In the bombykol biosynthetic pathway, bombykol is generated by the action of a unique bifunctional desaturase (desat) and a fatty-acyl reductase (FAR), both of which have been characterized as Bmpgdesat1 and PG- specific FAR [7], [8]. Similar to most lepidopteran sex pheromones, bombykol precursors are stored in cytoplasmic lipid droplets (LDs) in the form of triacylglycerols (TAGs), which rapidly accumulate on the day of eclosion [10]. During LD accumulation process, the PG-specific acyl-CoA-binding protein (ACBP) and fatty acid transport protein (FATP) were found to play vital roles [11], [12]. Most importantly, the PBAN receptor (PBANR), a G-protein coupled receptor (GPCR), was also identified in many lepidopteran species, including *B. mori* [13]. The roles of these genes in bombykol synthesis were further confirmed using the RNAi approach [9].

The molecular mechanisms of the PBAN signal transduction cascade were also elucidated in detail by the Matsumoto group [9]. Once PBAN binds with its receptor, it activates phospholipase C to release the two secondary messengers, inositol 1, 4, 5-triphosphate (IP_3_) and diacylglycerol (DAG). IP_3_ further promotes the release of Ca^2+^ from the endoplasmic reticulum (ER) [14]. The rapid release of Ca^2+^ stored in the ER depends on store operated channels (SOCs), a Ca^2+^ permeable cation channel activated by soluble IP_3_. Furthermore *in vivo* RNAi-mediated knockdown also confirmed that stromal interaction molecule 1 (STM1) and Orail, two essential components of SOCs, are involved in the PBAN signal transduction cascade [15].

LDs, which are stored in the cytoplasm, contain various TAGs and serve as a reservoir for the de novo synthesis of the bombykol precursor [10]. Before adult emergence, PG cells rapidly accumulate LDs. After adult emergence, PBAN triggers the hydrolysis and conversion of fatty acid to bombykol precursor. Recent studies in *B. mori* have shown that phosphorylation of *Bombyx* lipid storage droplet protein-1 (Lsd1) is essential for PBAN-mediated LD lipolysis. PBAN-mediated calcium influx activates a Ca^2+^/calmodulin- dependent protein kinase II (BmCaMKII), which phosphorylates Lsd1 at Ser and Thr residues [16]. Although numerous genes have been identified as essential for sex pheromone production, little is known about the regulation of TAG lipolysis in PG cells except that phosphorylation of Lsd1 is required for activation of TAG lipolysis. For example, the specific lipases that mediated TAG hydrolysis in this pathway are unknown. In the present study, the gene expression profiles of PGs at different developmental stages were compared using digital gene expression (DGE) data. These expression profiles provide an invaluable resource for identifying candidate genes associated with sex pheromone synthesis in *B. mori*, leading to the identification and verification of four lipases involved in sex pheromone synthesis.

## Results

### DGE library sequencing

**Figure 1:**
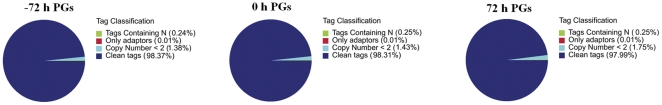
Different components of the raw tags in each sample. The percentages of clean tags, raw tags containing N, empty tags with adaptor only, and tags with copy number <2.

Three DGE libraries, including −72 h, 0 h and 72 h PGs, were sequenced using DGE methods. These DGE libraries generated over 5.7 million raw tags for each of the three libraries (Table 1). After filtering the adaptor sequences, low quality tags (tags with unknown nucleotide “N”), empty tags (only adaptor sequence) and tags with a copy number of one (probably be sequencing error), the three DGE libraries still generated over 5.6 million clean tags in each library, and the percentage of clean tags among raw tags in each library ranged from 97.9% to 98.37% (Fig. 1).

**Table 1:** Tag analysis statistics.

Summary	−72 h PGs	0 h PGs	72 h PGs
Raw Tag	6,137,521	5817084	5782637
Distinct raw tag	162,034	153748	192186
Clean tag	6037779	5718996	5666302
Distinct Clean tag	72774	65866	85348
All Tag Mapping to Gene	1,381,410	1,443,148	1,528,830
All Tag Mapping to Gene*	22.88%	25.23%	26.98%
Distinct All Tag Mapping to Gene	14868	13710	16851
Distinct All Tag Mapping to Gene*	20.43%	20.81%	19.74%
Unambiguous Tag Mapping to Gene	1342195	1402124	1471853
Unambiguous Tag Mapping to Gene*	22.23%	24.52%	25.98%
Distinct Unambiguous Tag Mapping to Gene	14575	13432	16490
Distinct Unambiguous Tag Mapping to Gene*	20.03%	20.39%	19.32%
All Tag-mapped Genes	5716	5174	5718
All Tag-mapped Genes**	39.09%	35.38%	39.10%
Unambiguous Tag-mapped Genes	5469	4932	5474
Unambiguous Tag-mapped Genes**	37.40%	33.73%	37.43%
Mapping to Genome	3867038	3420730	3190600
Mapping to Genome*	64.05%	59.81%	56.31%
Distinct Mapping to Genome	37360	33099	39740
Distinct Mapping to Genome*	51.34%	50.25%	46.56%
Unknown Tag	789331	855118	946872
Unknown Tag*	13.07%	14.95%	16.71%
Distinct Unknown Tag	20546	19057	28757
Distinct Unknown Tag*	28.23%	28.93%	33.69%

Notes:

* presents % of clean tag,

** indicates % of ref genes.

**Figure 2:**
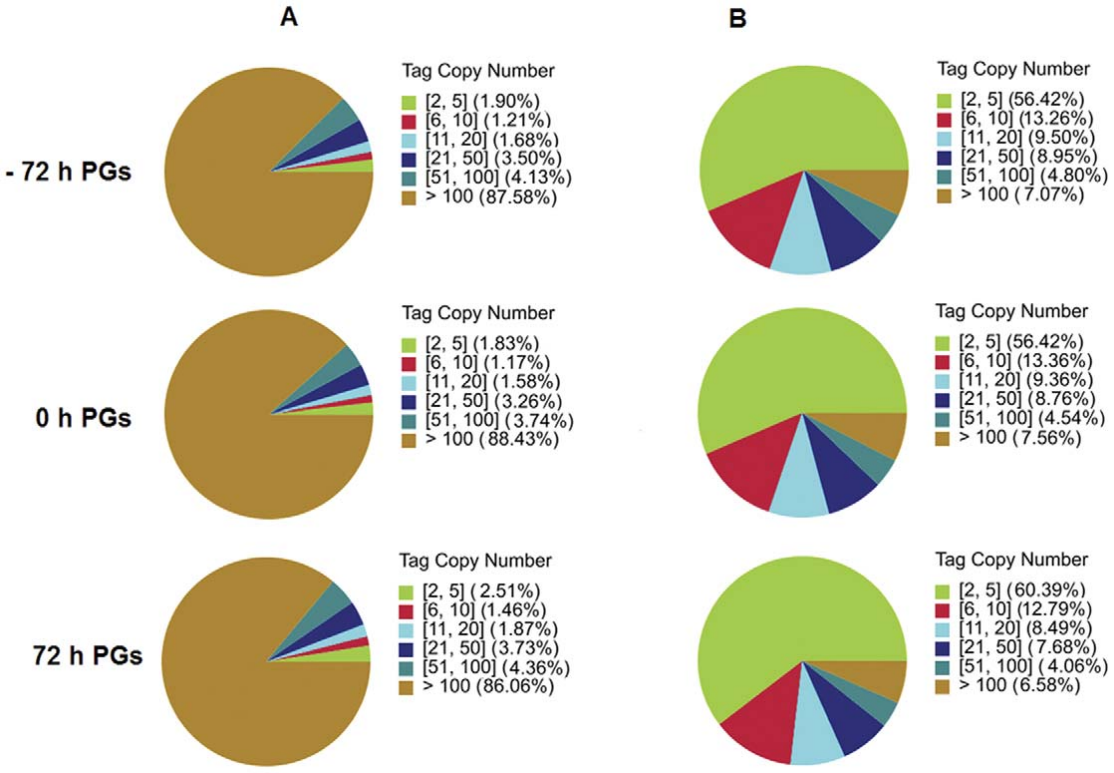
Distribution of total clean tags and distinct clean tags in each library. **A:** Distribution of total clean tags. **B:** Distribution of distinct clean tags.

To evaluate the normality of the DGE data, the distribution of clean tag expression was analyzed. As shown in Figure 2, the distribution of total clean tags and distinct clean tags in each library showed similar patterns; high-expression tags with more than 100 copy numbers comprised 85% of the total clean tags, but their distribution did not even reach 8.0% of the distinct clean tags, whereas low-expression tags with copy numbers smaller than 5 accounted for more than 56% of the distinct tag distribution.

### Mapping sequences to the reference genomic data

The sequencing of three libraries generated 72774, 65866, and 85348 distinct tags from −72 h, 0 h and 72 h PGs respectively. Among the distinct tags, the number that could be mapped to genes ranged from 13710 to 16851 (Table 1). To determine if the numbers of detected genes increase proportionally with sequenced tags, saturation analysis was performed (Fig. S1). When sequencing depths reached 1,000,000 total tags, the number of detected genes significantly dropped. When sequencing amount reach 2,000,000 or higher, the numbers of detected genes almost ceased to increase.

**Figure 3:**
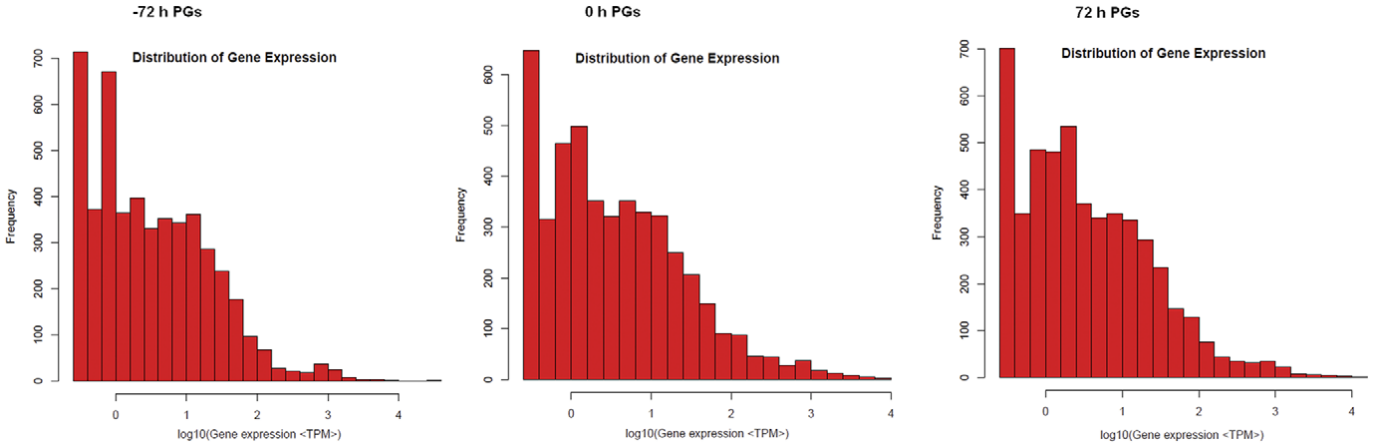
Distribution of gene expression. Gene expression level determined by calculating the number of unambiguous tags and then normalizing to TPM (transcript copies/million tags).

The level of gene expression was further analyzed by calculating the number of unambiguous tags for each gene and then normalizing to the number of transcripts per million tags (TPM). The results revealed that the majority of genes transcribed were represented by fewer than 10 copies and only a small proportion of genes were highly expressed (Fig. 3).

### Analysis of differentially expressed genes in the different developmental stages

**Figure 4:**
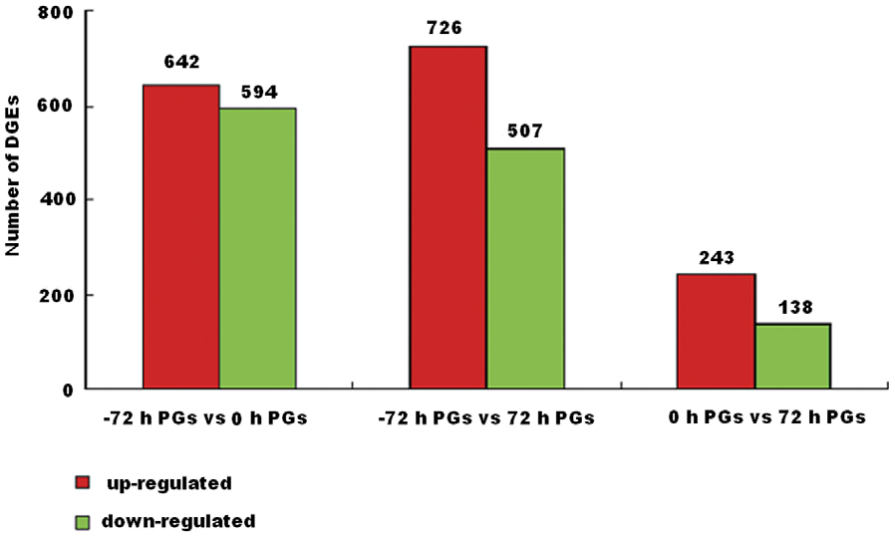
The numbers of differentially expressed genes at different developmental stages. Up- and down-regulated genes are summarized between −72 h and 0 h PGs, −72 h and 72 h PGs, and 0 h and 72 h PGs.

To identify genes showing a significant expression difference at different stages of PG development, the genes with differential expression between two samples were identified by using an algorithm developed by Audic *et al.* [17]. A P≤0.001 and absolute value of log2 ratio ≥1 were used as thresholds for significant differences in gene expression. The differences in gene expression patterns were analyzed for the pairs of −72 h and 0 h PGs, −72 h and 72 h PGs, and 0 h and 72 h PGs (Fig S2). A total 1236 genes were differentially expressed between −72 h and 0 h PG libraries (Fig. 4 and Table S1), with 642 up-regulated and 594 genes down-regulated. Gene Ontology (GO) classification analysis revealed that differentially expressed genes between −72 h and 0 h PGs correlated with alcohol metabolism, monosaccharide metabolism, amine metabolism, carbohydrate metabolism, chitin metabolism, amino sugar metabolism, glucosamine metabolism, or N-acetylglucosamine metabolism. Most of upregulated genes were significantly enriched in the KEGG orthology (KO) classification and associated with metabolic processes (Table S4).

Between −72 h and 72 h PGs libraries, 1233 genes were differentially expressed, including 726 upregulated and 507 downregulated genes (Fig. 4 and Table S2). According to GO classification, most of the up-regulated genes were associated with monosaccharide metabolism, alcohol metabolism or amine metabolism (Table S5).

When we compared the 0 h PG library with 72 h PG library, 381 differentially expressed genes were found, with 243 upregulated and 138 downregulated (Fig. 4 and Table S3). No significantly enriched genes were detected in GO and KO classification between 0 and 72 h PGs.

Most importantly, a serial of up-regulated genes in 0 h and 72 h were associated with the fatty acid synthesis and metabolism, consistent with the synthetic and released process of sex pheromone (Table S6).

### Gene expression and real-time PCR validation

**Figure 5:**
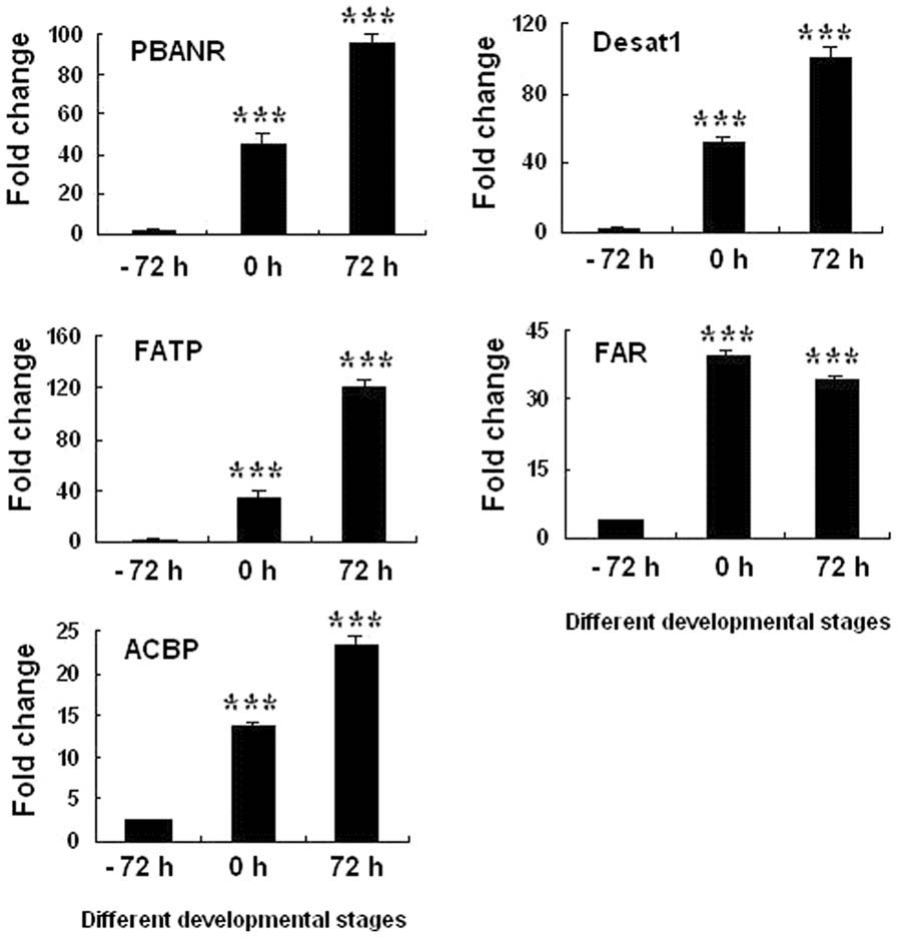
Temporal changes in relative expression levels of genes associated with pheromone synthesis in the PG of *B. mori* during pupal–adult development (the zero time point indicates the time of eclosion). PGs were collected at different developmental stages (−72, 0, and 72 h), and total RNA was extracted for real-time PCR analysis. The Rp49 gene was used as the housekeeping gene for normalization. The data represent the mean values ± SE of three biological replicates. Significance of pairwise comparisons (−72 h vs 0 h and −72 h vs 72 h) are marked with *** (p<0.001) as determined by the Student's *t*-test .

The PG is an important organ for sex pheromone production. Five sex pheromone synthesis signal genes, encoding for PBANR, FAR, ACBP, Desat1 and FATP, were found to be richly expressed at 0 h and 72 h PGs by DGE analysis (Table S1, Table S2, and Table S3). These genes play important roles in sex pheromone synthesis [4], [7], [8], [9], [11], [12] and could be used as marker genes for monitoring synthesis during development. Our real-time PCR results also confirm that these genes were richly expressed at 0 h and 72 h PGs, consistent with the DGE results (Fig. 5).

### Injection of dsRNA and *in vivo* bombykol analysis

**Figure 6:**
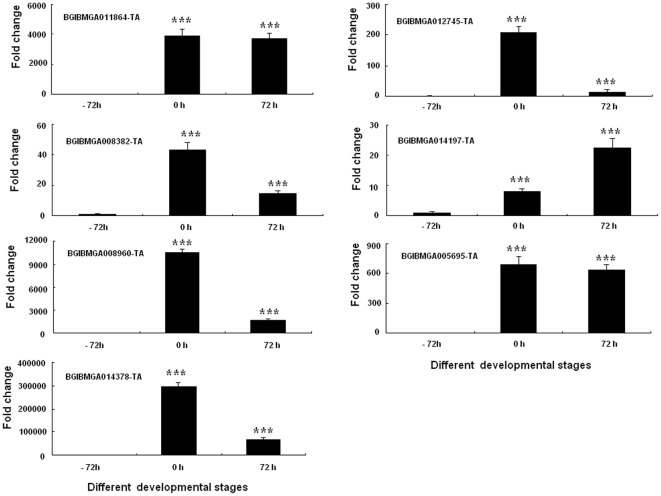
Differential expression of seven putative lipase genes at different developed stages. PGs were collected at different developmental stages (−72, 0, and 72 h), and total RNA was extracted for real-time PCR analysis. The Rp49 gene was used as the housekeeping gene for normalization. The data represent the mean values ± SE of three biological replicates. Significance of pairwise comparisons (−72 h vs 0 h and −72 h vs 72 h) are marked with *** (p<0.001) as determined by the Student's *t*-test .

**Figure 7:**
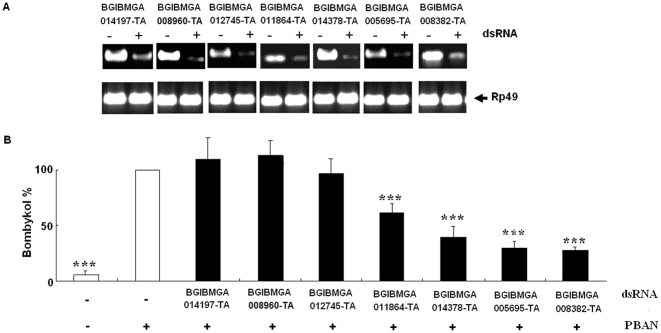
Effects of RNAi treatment on bombykol production. A: RNAi-induced reduction of seven putative lipase genes. RT-PCR was carried out using cDNA generated from the total RNA extracted from PGs of females injected with 20 µg dsRNAs for seven lipase genes and DEPC-treated nuclease-free water (Control). B: Effects of RNAi for seven lipase genes on bombykol production. Newly emerged females were decapitated and injected with double stranded RNAs. Decapitated females were injected with 5 pmol PBAN 30 h after dsRNA injection. Bombykol production was measured by GC/MS from PGs 90 min after injection of PBAN. Bars indicate the mean values ± S.D. for independent experimental animals (n> = 6). Statistically significant differences from the PBAN alone are denoted by *** (p<0.001) as determined by the Student's *t*-test .

Seven genes encoding putative lipases, BGIBMGA012745-TA, BGIBMGA011864-TA, BGIBMGA008382-TA, BGIBMGA014197-TA, BGIBMGA008960-TA BGIBMGA014378-TA and BGIBMGA005695-TA, were highly expressed at 0 h and 72 h PGs as revealed by DGE analysis (Fig. 6). To confirm the roles of these genes in PBAN stimulated sex pheromone synthesis, RNAi-mediated knockdown was performed. When 20 µg lipase dsRNA for each of the seven lipase genes was injected respectively into newly emerged and decapitated females, significant decrease in mRNA levels of all seven lipase genes was detected as indicated by PCR while injection of control had no effect on the developmental expression patterns of these mRNAs (Fig. 7A)

After successful reduction of lipase mRNAs by RNAi, PBAN-induced bombykol production was determined by GC/MS. The results demonstrated a significant reduction in bombykol production by RNAi. Bombykol production was reduced to 55% of control in the *BGIBMGA011864-TA* knockdown females, to 40% of control in the *BGIBMGA014378-TA* knockdown females, to 29% of control in the *BGIBMGA005695-TA* knockdown females, and to 27% of control in the *BGIBMGA008382-TA* knockdown females, suggesting that these genes play important roles in hydrolysis of TAGs (Fig. 7B).

## Discussion

Compared to conventional microarray-based methods, DGE generates sufficient data for the genome-wide analysis of gene expression and for the detection of every transcript present in a sample, including rare transcripts that would be otherwise lost in the background noise on microarrays. Indeed, most of the transcripts in a cell are rare, being present in only one to five copies [18], [19], [20]. In this report, we used the DGE technique to identify the genes involved in pheromone biosynthesis. For the deep-sequencing analysis of gene expression patterns, we first constructed three PG libraries from −72 h, 0 h and 72 h females. The DGE searched over 5.6 millions clean tags in each of the PGs library, which cover almost every transcript in the *B. mori* pheromone gland. We found a set of genes that were differentially expressed during PG development, thus defining a large set of putative genes involved in the developmental regulation of sex pheromone synthesis.

Bombykol is derived from acetyl-CoA through fatty acid synthesis and subsequent modification of the carbonyl carbon [1]. Our DGE analysis found that approximately 76%(92 of 126)of the up-regulated genes in the KO classification were associated with metabolic processes and were significantly up-regulated in the 0 h PGs, a key time point for sex pheromone biosynthesis and release (Table S4). Most of these up-regulated genes play essential roles in fatty acid biosynthesis and metabolism processes (Table S6). For example, the transcript *BGIBMGA008049-TA* encodes a diacylglycerol acyltransferase, which catalyzes the final step in TAG biosynthesis. Most importantly, this is the rate-limiting step in TAG biosynthesis [21]. *BGIBMGA005057-TA* encodes a glycerol-3-phosphate acyltransferase, which is the enzyme catalyzing the initial step of de novo TAG synthesis in the glycerol phosphate pathway [22]. In addition, the transcripts *BGIBMGA014378-TA*, *BGIBMGA008960-TA*, *BGIBMGA011864-TA*, *BGIBMGA012745-TA*, *BGIBMGA005695-TA*, *BGIBMGA008382-TA* and *BGIBMGA014197-TA* encode putative lipases, which play essential roles in TAG lipolysis. These findings are consistent with the process of sex pheromone biosynthesis and release, which involve fatty acid biosynthesis and metabolism. All the genes screened in the present DGE analysis are expected to provide valuable information regarding the synthesis and release of sex pheromone.

Great strides have been made in recent years to elucidate the mechanism of sex pheromone synthesis. Many associated genes, such as FATP, PBANR, FAR, ACBP, and Desat1, have been identified as essential components in bombykol synthesis [4], [7], [8], [9], [11], [12]. The functions of these genes were comprehensively described; for example, FATP functions as a transporter that facilitates the uptake of extracellular long-chain fatty acids across the plasma membrane [11], whereas ACBP functions by protecting fatty acyl-CoA esters from hydrolysis, thus ensuring an adequate pool of fatty acid precursors for bombykol synthesis [11], [12]. In our DGE study, FATP, PBANR, FAR, ACBP, and Desat1 were found to be dramatically upregulated in 0 h and 72 h PGs compared to the levels present in the −72 h PGs. Real-time PCR results further confirmed that these genes were richly expressed by 0 h and 72 h PGs, consistent with the results of previous reports [4], [7], [8], [9], [11], [12].

In contrast to *Helicoverpa* species, *Bombyx* females release sex pheromone immediately after emergence. Thus, before emergence as an adult, female must accumulate sufficient amounts of fatty acid precursors for sex pheromone synthesis and release. In *Bombyx*, LDs containing TAGs are the reservoir for the synthesis of bombykol precursor. Moreover, lipolysis of TAGs is known to occur under the PBAN stimulation following emergence. Recent studies have demonstrated that the BmLsd1 protein, a homolog of the mammalian perilipin, was activated by CaMKII-mediated phosphorylation rather than by protein kinase A [23], [24]. The BmLsd1 protein is located on the LD surface of fat cells where it prevents lipolysis under basal condition. Once activated by phosphorylation upon PBAN stimulation, the lipolysis of TAG will occur by lipase activity. The activation of BmLsd1 is a key step in the activation of the lipolytic cascade. In *Bombyx*, TAGs in the PG must undergo lipolysis for release of the sex pheromone precursor. Our DGE data identified seven putative lipase genes that were dramatically upregulated in 0 h PGs, a key time point for sex pheromone production in *Bombyx*. Further RNAi-mediated knockdown of these putative lipases demonstrated that four of the seven genes are essential for bombykol synthesis.

In mammals, three lipases which have been implicated in the complete hydrolysis of TAGs, are adipose triglyceride lipase (ATGL), hormone sensitive lipase (HSL), and monoacylglycerol lipase (MAGL) [25]. ATGL is a rate-limiting enzyme for hydrolyzing TAGs to generate diacylglycerols (DAGs) and free fatty acids, whereas HSL acts by hydrolyzing a variety of acylesters, including TAGs, DAGs, and MAGs and is the rate-limiting enzyme for DAG catabolism. Finally, MAGL cleaves MAGs into glycerol and free fatty acids [26], [27], [28]. In fact, different TAG lipases are required to hydrolyze different TAGs into unique DAGs, like 1, 3-DAG, 2-DAG, and 1, 2-DAG [28].

In *Bombyx*, bombykol is generated, after PBAN stimulation, from fatty acyl precursors in the TAGs. These fatty acyl precursors are limited to five unsaturated C_16_ and C_18_ fatty acyl groups, including Δ11-hexadecenoate, Δ10, 12-hexadecadienoate, Δ9-octadecenoate, Δ9, 12-octadecadienoate and Δ9, 12, 15-octadecatrienoate, which form various TAGs [10]. This explains the need for the four lipase genes in *B. mori* for complete hydrolysis of TAGs, as detected by RNAi-mediated knockdown, although the substrates of these lipases are still unknown, even in mammals. Surprisingly, the remaining 3 lipases have no effect on sex pheromone synthesis, despite being significantly repressed at the mRNA level (Fig. 6). Most importantly, recent studies have shown that PBAN apparently regulates the activity of pheromone-producing enzymes through receptor-mediated membrane transduction. Thus regulation of these lipases occurs at both the transcript and protein levels [4], [16]. The mechanisms by which these four lipases show PBAN-induction activation and the functions of the remaining three lipases in the PGs of *B. mori* require further investigation that is currently underway.

## Materials and Methods

### Insects

Silkworm *B. mori* (Zhenzhu×Chunlei) eggs were obtained from the Sericultural Research Institute of NeiXiang, Henan Academy of Agricultural Sciences. The insects were reared on mulberry leaves at 26°C under a 16 h light/8 h dark cycle.

Pupae were separated by gender, and adult males and females emerged in separate cages until tissue dissection.

### Chemicals

*B. mori* PBAN was synthesized by Sangon Biotech (Shanghai) Co., Ltd. The main sex pheromone component, Bombykol, was a gift from Shogo Matsumoto (RIKEN, Advanced Science Institute, Japan). Bombykol was used as the internal standard quantification for gas chromatography- mass spectrometry (GC/MS).

### Sample collection and RNA extraction

PGs were dissected at different developmental stages (−72 h, 0 h, and 72 h) and immediately placed at −80°C for later use.

Total RNA was extracted from the collected samples using Trizol reagent (Invitrogen) following the manufacturer's instructions. RNA concentration was further determined by measuring the absorbance at 260 nm on a spectrophotometer.

### DGE library preparation and sequencing

Three DGE libraries (−72 h, 0 h, and 72 h PGs) were prepared by using the Illumina Gene Expression Sample Prep Kit. Briefly, mRNA was purified using Oligo(dT) magnetic beads from 6 µg total RNA. The first and second-strand cDNA were synthesized while the RNA was bound to the beads. The bead-bound cDNA was subsequently digested with restriction enzyme *Nla*III, which recognizes and cuts CATG sites. cDNA fragments with 3′ ends were purified through bead precipitation, and the Illumina adaptor 1 was added to the sticky 5′ end of these cDNA fragments. The junction of Illumina adaptor 1 and the CATG site is the recognition site of *Mme*I, which cuts at 17 bp downstream of the CATG site. Twenty-one base-pair tags containing adaptor 1 were further produced. After removing the 3′ fragments by magnetic bead precipitation, Illumina adaptor 2 was ligated to the 3′ ends of tags to yield tags with different adaptors at both ends. A tag library was obtained. The library was further amplified by PCR for 15 cycles, and 85 bp fragments were purified by 6% TBE PAGE gel electrophoresis. The single-chain nucleotides were fixed onto the Illumina Sequencing Chip (flowcell) after denaturation. Adaptor 1 was used as the sequencing primer. Each tunnel will generate millions of raw reads with sequencing length of 35 bp.

### Aligning DGE tags to reference genomic data

Sequencing-received raw image data were transformed by base calling into sequence data, called raw data or raw reads. To map the DGE tags, the raw data were filtered after data processing, including removal of adaptor sequences, low quality tags (tags with unknown nucleotide “N”), empty tags (only adaptor sequence), and tags with a copy number of 1 (probably from sequencing errors). All possible CAGT+17 nucleotide tags were created by using the *Bombyx* genomic database and other NCBI data. All clean tags were mapped to the reference sequences and only 1 bp mismatch was allowed. Clean tags mapped to reference sequences from multiple genes were filtered. The remaining clean tags were designated as unambiguous clean tags. The number of unambiguous clean tags for each gene was calculated and then normalized to TPM (number of transcripts per million clean tags) [20], [29].

### Evaluation of DGE libraries

To compare the gene expression in PGs at different developmental stages, the tag frequency in each DGE library was statistically analyzed according to the method described by Audic *et al*. [17]. The false discovery rate (FDR) was used to determine the threshold P-value in multiple tests. A FDR<0.001 and an absolute value of the log2 ratio>1 were used as the thresholds to determine significant differences in gene expression.

Differentially expressed genes were chosen for further GO and KO enrichment analysis based on hypergeometric tests. The calculating formula was
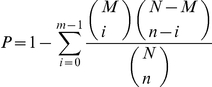
Where N represents the number of genes with GO/KO annotation, n represents the number of DGEs in N, M represents the number of genes in each GO/KO term, and m represents the number expressed gene in each GO/KO term. A corrected P-value<0.05 were selected as a threshold for significant enrichment of the gene sets. In contrast, for KO enrichment analysis, FDR<0.05 was used as the threshold to determine significant enrichment of the gene sets

### Real-time PCR

Total RNA (1 µg) from each sample was reverse transcribed to generate the first-strand cDNA using the PrimeScript RT reagent kit with gDNA Eraser (TaKaRa). The primers designed for real-time PCR analysis are listed in Table S7. *Bombyx* ribosomal protein 49 (rp49) gene was used as an internal gene for normalization. Real-time PCR was carried out using SYBR Green Supermix (TaKaRa) according the manufacturer's instructions. The thermal cycle conditions used in the real-time PCR were 95°C for 10 min, followed by 40 cycles of 95°C for 15 sec and 60°C for 1 min. The specificity of the SYBR green PCR signal was confirmed by melting curve analysis and agarose gel electrophoresis. The mRNA expression was quantified using the comparative CT (Cross Threshold, the PCR cycle number that crosses the signal threshold) method [30]. The CT of the RP49 gene was subtracted from the CT of the target gene to obtain ΔCT. The normalized fold changes of the target gene mRNA expression were expressed as 2^−ΔΔCT^, where ΔΔCT is equal to ΔCT_treated sample_−ΔCT_control_.

### Double-stranded RNA (dsRNA) synthesis

Double stranded RNAs were synthesized using the MEGAscript RNAi kit (Ambion) according to the manufacturer's instructions. PCR was performed using gene-specific primers containing T7 polymerase sites. All the primer sets are listed in Table S8. PCR was performed at 94°C for 3 min, followed by 35 cycles of 94°C for 1 min, 56°C for 1 min, and 72°C for 1 min, and a final elongation at 72°C for 10 min. Template DNA and single-stranded RNA were removed from the transcription reaction by DNase and RNase treatments. Double stranded RNA was purified using MEGAclearTM columns (Ambion) and eluted in diethyl pyrocarbonate (DEPC)-treated nuclease-free water. The dsRNA concentrations were measured using a biophotometer (Eppendorf). Our previous experiments confirmed that either dsGFP RNA or ddH_2_O were suitable negative controls. In the present study, DEPC-treated nuclease-free water was used as a negative control.

The effects of RNAi on the transcript expression were analyzed by using RT-PCR. The primers used in this experiment are shown in Table S9.

### Injection of dsRNA and *in vivo* bombykol analysis

The newly emerged females were decapitated followed by injection of 20 µg lipase dsRNA as described [7]. Control females were injected with DEPC-treated nuclease-free water. The females were maintained for 30 h under normal conditions and then injected with either 5 pmol *B. mori* PBAN in PBS or PBS alone. The PGs were dissected 90 min after injection and dissolved in hexan.

Bombykol accumulation was measured by GC/MS (Trace GC Ultra Trace DSQ; MS-Thermo Scientific DSQ II) equipped with a 30 m capillary column (RTX-5SILMS, Restek, 0.25 mm diameter). Each sample contains a pooled hexane extract from 6 or more *B. mori* PGs. Each sample was then subjected to GC/MS analysis.

## Supporting Information

Figure S1 Saturation analysis of sequencing.
(TIF)

Figure S2 Gene expression level in PGs during different developmental stages. “Not DEGs” indicates “not detected expression genes”. X-axis and Y-axis present log10 of the transcript per million of differentially developed stages of PGs. P< = 0.001 and absolute value of log2 > = 1 were used as the thresholds.
(TIF)

Table S1 Differentially expressed genes between −72 h and 0 h PGs.
(XLS)

Table S2 Differentially expressed genes between −72 h and 72 h PGs.
(XLS)

Table S3 Differentially expressed genes between 0 h and 72 h PGs.
(XLS)

Table S4 Gene set enrichment analysis comparing −72 h and 0 h PGs.
(DOC)

Table S5 Gene set enrichment analysis comparing −72 h and 72 h PGs.
(DOC)

Table S6 List of upregulated genes regarding the fatty acid synthesis and metabolism in 0 h and 72 h PGs.
(DOC)

Table S7 Primers used in real-time PCR for validation of the differentially expressed sex pheromone synthesis genes.
(DOC)

Table S8 Primers used in dsRNA synthesis.
(DOC)

Table S9 Primers used for validation of the RNAi effect.
(DOC)
