# Supplementary figures and images for: Identification of Lipases Involved in PBAN Stimulated Pheromone Production in Bombyx mori Using the DGE and RNAi Approaches
Source: PLoS One. 2012 Feb 16;7(2):e31045. doi: 10.1371/journal.pone.0031045 (PMC3281041; doi:10.1371/journal.pone.0031045)

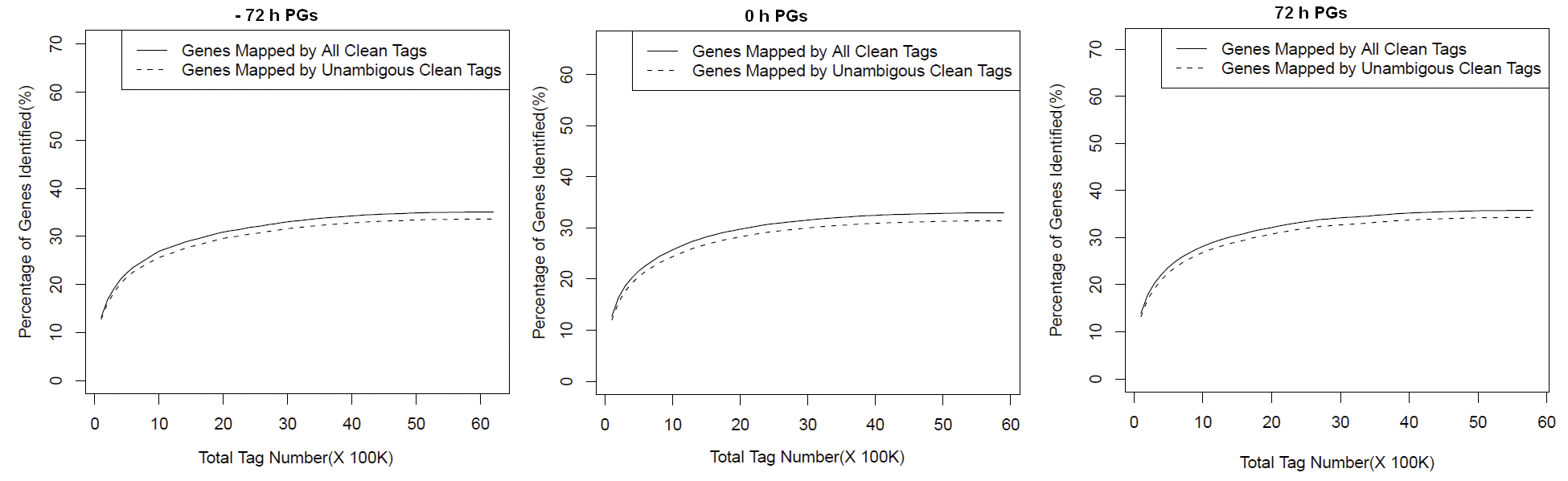

Supplement: Figure S1 — Saturation analysis of sequencing. (TIF) [file pone.0031045.s001.tif]

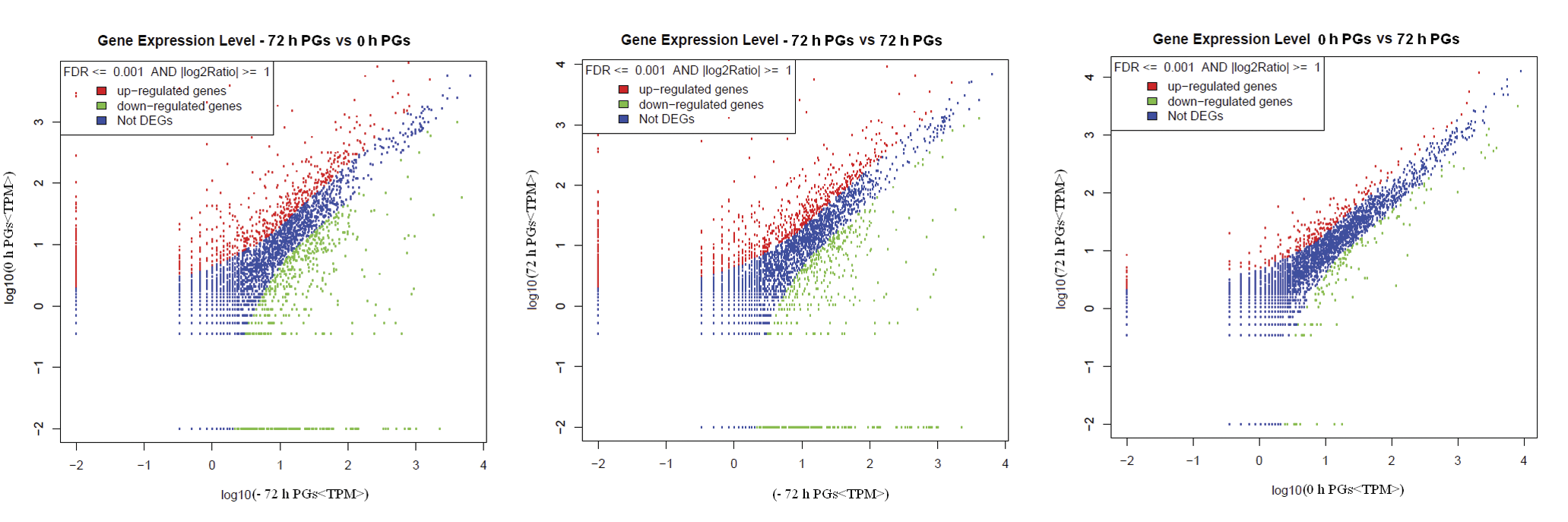

Supplement: Figure S2 — Gene expression level in PGs during different developmental stages. “Not DEGs” indicates “not detected expression genes”. X-axis and Y-axis present log10 of the transcript per million of differentially developed stages of PGs. P< = 0.001 and absolute value of log2 > = 1 were used as the thresholds. (TIF) [file pone.0031045.s002.tif]
