# Supplementary material for: Identification of Lipases Involved in PBAN Stimulated Pheromone Production in Bombyx mori Using the DGE and RNAi Approaches
Source: PLoS One. 2012 Feb 16;7(2):e31045. doi: 10.1371/journal.pone.0031045 (PMC3281041; doi:10.1371/journal.pone.0031045)
Supplement: Table S4 — Gene set enrichment analysis comparing −72 h and 0 h PGs. (DOC) [file pone.0031045.s006.doc]

**Supplemental Table S4.** Gene set enrichment analysis comparing -72 h and 0 h PGs

| **Category** | **Subcategory** | **P-value** | **Total** | **Up-regulation** | **Down-regulation** |
| --- | --- | --- | --- | --- | --- |
| **Biological Process** | alcohol metabolic process | 2.62E-03 | 20 | 11 | 9 |
|  | monosaccharide metabolic process | 3.33E-03 | 17 | 9 | 8 |
|  | amine metabolic process | 1.07E-02 | 23 | 3 | 20 |
|  | carbohydrate metabolic process | 1.81E-02 | 34 | 11 | 23 |
|  | metabolic process | 2.04E-02 | 170 | 92 | 78 |
|  | chitin metabolic process | 3.85E-02 | 9 | 0 | 9 |
|  | amino sugar metabolic process | 3.85E-02 | 9 | 0 | 9 |
|  | glucosamine metabolic process | 3.85E-02 | 9 | 0 | 9 |
|  | N-acetylglucosamine metabolic process | 3.85E-02 | 9 | 0 | 9 |
| **Cellular Component** | cytoplasm | 2.40E-04 | 89 | 56 | 33 |
| **Molecular Function** | carbohydrate binding | 8.20E-04 | 14 | 3 | 11 |
|  | catalytic activity | 4.40E-03 | 170 | 92 | 78 |
|  | chitin binding | 9.60E-03 | 8 | 0 | 8 |
|  | oxidoreductase activity | 2.00E-02 | 36 | 25 | 9 |
|  | pattern binding | 4.30E-02 | 8 | 0 | 8 |
|  | polysaccharide binding | 4.30E-02 | 8 | 0 | 8 |
