# Supplementary material for: Identification of Lipases Involved in PBAN Stimulated Pheromone Production in Bombyx mori Using the DGE and RNAi Approaches
Source: PLoS One. 2012 Feb 16;7(2):e31045. doi: 10.1371/journal.pone.0031045 (PMC3281041; doi:10.1371/journal.pone.0031045)
Supplement: Table S5 — Gene set enrichment analysis comparing −72 h and 72 h PGs. (DOC) [file pone.0031045.s007.doc]

**Supplemental Table S5.** Gene set enrichment analysis comparing -72 h PGs and 72 h PGs

| **Category** | **Subcategory** | **P-value** | **Total** | **Up-regulation** | **Down-regulation** |
| --- | --- | --- | --- | --- | --- |
| **Biological Process** | monosaccharide metabolic process | 3.2E-03 | 17 | 7 | 10 |
|  | alcohol metabolic process | 1.0E-02 | 19 | 9 | 10 |
|  | amine metabolic process | 1.4E-02 | 11 | 2 | 9 |
|  | chitin metabolic process | 3.5E-02 | 9 | 0 | 9 |
|  | amino sugar metabolic process | 3.5E-02 | 9 | 0 | 9 |
|  | glucosamine metabolic process | 3.5E-02 | 9 | 0 | 9 |
|  | N-acetylglucosamine metabolic process | 3.5E-02 | 9 | 0 | 9 |
| **Cellular Component** | cytoplasm | 4.32E-07 | 103 | 74 | 29 |
|  | [cytoplasmic part](http://amigo.geneontology.org/cgi-bin/amigo/go.cgi?action=query&view=query&query=GO:0044444&search_constraint=terms) | 7.02E-06 | 81 | 60 | 21 |
|  | [lipid particle](http://amigo.geneontology.org/cgi-bin/amigo/go.cgi?action=query&view=query&query=GO:0005811&search_constraint=terms) | 1.2E-02 | 14 | 12 | 2 |
| **Molecular Function** | carbohydrate binding | 3.80E-03 | 13 | 2 | 11 |
|  | chitin binding | 7.8E-03 | 8 | 0 | 8 |
|  | pattern binding | 3.5E-02 | 8 | 0 | 8 |
|  | polysaccharide binding | 3.5E-02 | 8 | 0 | 8 |
