# Supplementary material for: Identification of Lipases Involved in PBAN Stimulated Pheromone Production in Bombyx mori Using the DGE and RNAi Approaches
Source: PLoS One. 2012 Feb 16;7(2):e31045. doi: 10.1371/journal.pone.0031045 (PMC3281041; doi:10.1371/journal.pone.0031045)
Supplement: Table S6 — List of upregulated genes regarding the fatty acid synthesis and metabolism in 0 h and 72 h PGs. (DOC) [file pone.0031045.s008.doc]

**Table S6. List of the up-regulated Genes in 0 h and 72 h related to fatty acid synthesis and metabolism**

**Transcript Putative gene names**

BGIBMGA014378-TA lipase

BGIBMGA008960-TA lipase

BGIBMGA011864-TA lipase

BGIBMGA012745-TA lipase

BGIBMGA005695-TA lipase

BGIBMGA008382-TA lipase

BGIBMGA014197-TA lipase

BGIBMGA012925-TA phopholipase A2

BGIBMGA008049-TA diacylglycerol o-acyltransferase

BGIBMGA005057-TA glycerol-3-phosphate acyltransferase

BGIBMGA008524-TA AMP dependent coa ligase

BGIBMGA003156-TA methylacyl-coa racemase

BGIBMGA003362-TA phosphocholine cytidylyltransferase 1

BGIBMGA001323-TA short-chain dehydrogenease/reductase

BGIBMGA000511-TA 3-hydroxyacyl-CoA dehydrogenase

BGIBMGA006083-TA carbonyl reductase

BGIBMGA002886-TA short-chain dehydrogenase

BGIBMGA008902-TA short-chain dehydrogenase,

BGIBMGA011108-TA short-chain dehydrogenase,

BGIBMGA008440-TA aldehyde oxidase

BGIBMGA008439-TA aldehyde oxidase

BGIBMGA002457-TA aldehyde dehydrogenase

BGIBMGA007361-TA 3-hydroxyacyl-CoA dehydrogenase

BGIBMGA003709-TA long-chain acyl-CoA synthetases

BGIBMGA000715-TA acyl-CoA dehydrogenase

BGIBMGA007409-TA 3-hydroxyacyl-coa dehyrogenase

BGIBMGA011029-TA acetoacetyl-CoA thiolase

BGIBMGA001966-TA aldehyde dehydrogenase isoform 1

BGIBMGA013593-TA perilipin

BGIBMGA012890-TA fatty acid binding protein

BGIBMGA000069-TA fatty acid desaturase

BGIBMGA006185-TA fatty acid transport protein

BGIBMGA010458-TA fatty-acyl reductase

BGIBMGA007767-TA glycerophosphoryl diester phosphodiesterase

BGIBMGA000858-TA acyl_transf_3

BGIBMGA001219-TA choline kinase activity

BGIBMGA013254-TA aldo-keto reductase

BGIBMGA004229-TA esterase
