# Supplementary material for: Identification of Lipases Involved in PBAN Stimulated Pheromone Production in Bombyx mori Using the DGE and RNAi Approaches
Source: PLoS One. 2012 Feb 16;7(2):e31045. doi: 10.1371/journal.pone.0031045 (PMC3281041; doi:10.1371/journal.pone.0031045)
Supplement: Table S7 — Primers used in real-time PCR for validation of the differentially expressed sex pheromone synthesis genes. (DOC) [file pone.0031045.s009.doc]

Table S7. List of primers used in real-time PCR analysis

Gene Forward primer (5′–3′) Reverse primer (5′–3′)

Desat1 gggaactttggtattacag tcgtagcattatgagggtctg

FAR agaaacttgcgtattcgtgtc ttagtttgaccgaagcggctgag

PBANR tcttagggaatacaagcacat atagcaatgtatcgctccatagt

FATP atagtggacattgaaggcaca tcttcaactcctaagggcacgt

ACBP tgaccaagccgcagccaacgt tgaggccgatggaagctatg

Rp49 CAGGCGGTTCAAGGGTCAATAC TGCTGGGCTCTTTCCACGA

Desat1: Acyl-CoA Desaturase; FAR: Fatty Acyl Reductase; PBANR: PBAN Receptor; FATP: Fatty Acid Transport Protein; ACBP: Acyl-CoA Binding Protein; Rp49: ribosomal protein 49
