# Supplementary material for: Identification of Lipases Involved in PBAN Stimulated Pheromone Production in Bombyx mori Using the DGE and RNAi Approaches
Source: PLoS One. 2012 Feb 16;7(2):e31045. doi: 10.1371/journal.pone.0031045 (PMC3281041; doi:10.1371/journal.pone.0031045)
Supplement: Table S8 — Primers used in dsRNA synthesis. (DOC) [file pone.0031045.s010.doc]

**Table S8 List of primers used in dsRNA synthesis**

Gene Forward primer (5′–3′) Reverse primer (5′–3′)

| BGIBMGA014378-TA | GATCACTAATACGACTCACTATAGGGAGAAAAGACTGATGTATGCTCGT | GATCACTAATACGACTCACTATAGGGAGAtgttgactagtttatcaaca |
| --- | --- | --- |
| BGIBMGA005695-TA | GATCACTAATACGACTCACTATAGGGAGATGTCGCCAACGCGGCAAGA | GATCACTAATACGACTCACTATAGGGAGATCGCGAGTTCCCATGTGAC |
| BGIBMGA012745-TA | GATCACTAATACGACTCACTATAGGGAGAAGCACCGGCAGTCCACATTG | GATCACTAATACGACTCACTATAGGGAGAGTTGGCATTCCTCCCCCATA |
| BGIBMGA008960-TA | GATCACTAATACGACTCACTATAGGGAGATGATCATTGCTGTCCACGT | GATCACTAATACGACTCACTATAGGGAGATCCGCTCCTAATGAGAACC |
| BGIBMGA011864-TA | GATCACTAATACGACTCACTATAGGGAGATGTCCAGTGAAGAATCTCCT | GATCACTAATACGACTCACTATAGGGAGAAGGATCAAGGCCCGTGGCT |
| BGIBMGA008382-TA | GATCACTAATACGACTCACTATAGGGAGACTTATCAGCAAATGTTGGAG | GATCACTAATACGACTCACTATAGGGAGATCATGCCCCCATTTGGGTA |
| BGIBMGA014197-TA | GATCACTAATACGACTCACTATAGGGAGAAGTCTTGGGGGTCATCTGGT | GATCACTAATACGACTCACTATAGGGAGAAGGCTTCAAACAAATCAACC |
