# Supplementary material for: Identification of Lipases Involved in PBAN Stimulated Pheromone Production in Bombyx mori Using the DGE and RNAi Approaches
Source: PLoS One. 2012 Feb 16;7(2):e31045. doi: 10.1371/journal.pone.0031045 (PMC3281041; doi:10.1371/journal.pone.0031045)
Supplement: Table S9 — Primers used for validation of the RNAi effect. (DOC) [file pone.0031045.s011.doc]

**Table S9 List of primers used in validation of RNAi effect**

Gene Forward primer (5′–3′) Reverse primer (5′–3′)

BGIBMGA014378-TA AAAGACTGATGTATGCTCGT TGATAGATTGTATGGCTTCAC

BGIBMGA005695-TA AGACCCACTCGCTCATCTC TCTTATGTTTGGCATCCAGAC

BGIBMGA012745-TA GCTGGACTTCAACGGTCTC AGCCAGCGTTCGCGAGGGA

BGIBMGA008960-TA ATGTCAGCCCCGGTGGGGGA TCCGCTCCTAATGAGAACC

BGIBMGA008382-TA TGTATTCTGTCAACCTGCGAT TAGCCCGAACGGTGATGAT

BGIBMGA014197-TA GACGATTTGGAGTTAGTCG GGCGATACCAAAGCCGTCTAT

BGIBMGA011864-TA TGGGTGACAGCGATTTATC ACGAGTCGCTTCCTTTCAC

Bmrp49 CAGGCGGTTCAAGGGTCAATAC TGCTGGGCTCTTTCCACGA
